# Physician Assisted Death for Psychiatric Suffering: Experiences in the Netherlands

**DOI:** 10.3389/fpsyt.2022.895387

**Published:** 2022-06-20

**Authors:** SMP van Veen, GAM Widdershoven, ATF Beekman, N. Evans

**Affiliations:** ^1^Department of Psychiatry, Amsterdam University Medical Center, Amsterdam, Netherlands; ^2^Department of Ethics, Law and Humanities, Amsterdam University Medical Center, Amsterdam, Netherlands; ^3^113 Suicide Prevention, Amsterdam, Netherlands

**Keywords:** Euthanasia (active voluntary), physician assisted death, psychiatry, irremediability, Netherlands, medical aid in dying, competence

## Abstract

Physician assisted death (PAD) for patients with a psychiatric disorder is a controversial topic of increasing relevance, since a growing number of countries are allowing it. General requirements for PAD include that patients possess decision-making capacity to decide on PAD and that their suffering is unbearable and irremediable. In the Netherlands PAD has been eligible for patients with psychiatric disorders since the 1990s, making it one of the few countries that can offer insights on the practice from real life experience. Much of the literature describing these experiences is only available in Dutch. This article aims to make this knowledge more widely available and provide a comprehensive overview of the experience with PAD for psychiatric suffering in the Netherlands. First, the history of PAD for patients suffering from a psychiatric disorder is described. Second, an overview of relevant rules and regulations governing the practice is given. Third, an overview is provided of the scarce epidemiological data. Finally, we will discuss two major clinical challenges; establishing irremediability and decision-making capacity.

## Introduction

Physician assisted death (PAD) is a controversial topic of increasing relevance. Public acceptance appears to be on the rise and countries around the world are legalizing a form of assisted death or are debating it. An even more controversial topic is whether to allow PAD for patients with a psychiatric disorder, which is possible in only a few countries, such as the Netherlands, Belgium, Luxembourg, Switzerland and soon also Canada (1). Many countries choose to exclude psychiatric suffering as a justified ground for PAD. Either explicitly by demanding that the cause of suffering is physical, or more often implicitly by installing a criterium that death has to be foreseeable. This exclusion is a topic of academic and societal debate (1). In the Netherlands, PAD is possible for patients who have a voluntary and well-considered death wish and who suffer from a medical or psychiatric condition that is unbearable and irremediable. Although PAD due to psychiatric suffering has been possible since the 1990s, until 2010 only a few cases were reported yearly. However, since 2011 there has been a remarkable increase, with 115 cases being recorded in 2021 - the most recent year for which figures are available (2). This makes the Netherlands one of the few countries in the world that can offer insights on the practice from real life experience. Because of this exceptional position, patients, clinicians, activists, ethicists and policymakers from around the world often refer to the Netherlands when debating PAD for psychiatric suffering, sometimes cherry-picking facts and figures to support their own standpoints.

In the meantime, many sources that accurately describe the situation in the Netherlands are only available in the Netherlands, and are largely unavailable to international readers.^1^ This increases the risk of misunderstandings and hampers a rational and informed debate about an already complex topic. This article aims to bridge this knowledge gap by giving a comprehensive overview of the Dutch experience with PAD for patients with a psychiatric disorder. The developments in other countries that allow PAD are described elsewhere (1). Here we will describe the history, rules and regulations, and epidemiology of PAD for psychiatric suffering in the Netherlands. After that we will discuss the issues that are currently being debated.

## History

The current debate about PAD in the Netherlands originates in the 1970s. General practitioners raised awareness of the tension they experienced between the duty to alleviate suffering and the duty to preserve life. Some admitted to occasionally helping people to die at their own request. Anecdotal evidence suggests that these cases mainly concerned cancer patients with a terminal diagnosis and limited life expectancy. Persons who wanted to die because of psychiatric suffering were not yet discussed. A growing societal movement advocated for more transparency and regulation of assisted death, culminating in the establishment of the ‘Voluntary Euthanasia Foundation’, in 1973. A member of this foundation, Mrs. Wertheim, helped an older lady to die at her request. She was charged with assisted suicide in 1983 and sentenced to 6 months of suspended imprisonment. In the verdict, however, the judge gave the first outline for due diligence requirements that could be grounds for acquittal. One of these was that a physician should be involved. The judge also remarked that psychiatric suffering can cause unbearable suffering and that the patient does not have to be close to death in order to be eligible for PAD.

The first psychiatrist to publish about PAD for psychiatric suffering was Dr. F. van Ree. In 1982, he wrote a commentary in the Dutch Journal of Psychiatry describing three patients with persistent suicidal ideations. Two eventually committed suicide in a humane manner, unassisted but also unhindered by the mental health professionals, and one patient eventually recovered after long and involuntary clinical treatment. In his view, the cases demonstrated that PAD should be regarded as a last resort. In a 1983 article, van Ree is the first to remark that accurate diagnosis and prognosis are challenging in psychiatry and to argue that due expertise by psychiatrists is essential during a PAD-procedure. Or as he put it: ‘anyone who has never seen how people can recover from a very deep, vital depression and a seemingly hopeless state, cannot make a sound judgment about assisted suicide' (3).

At the start of the 1990s, the Dutch Psychiatry Association and the Royal Dutch Medical Association both issued a statement that if PAD is allowed for somatic suffering, it should also be allowed for psychiatric suffering (4). This viewpoint was confirmed in 1994 by the ruling of the supreme court in the Chabot case. This case received much national and international attention and formed the basis of numerous publications about PAD for psychiatric suffering in the following years. It concerned a 50-year-old woman with a depressed mood who wanted to end her life after both her sons had died. Over a period of 2 months, psychiatrist Chabot let her stay in his private guesthouse and met with her to discuss her death wish. She refused psychotherapeutic or psychopharmaceutical treatment. After establishing that she was mentally competent and that there were no treatment options left, he assisted in her death by prescribing medication. Although he later stated that he consulted seven colleagues, none of these other psychiatrists examined the woman themselves. Chabot was found guilty of assisted suicide, but no penalty was imposed. In the ruling the judges made clear that, although extreme caution is advised, psychiatric suffering can be a justified ground for PAD. Furthermore, this ruling inspired the practice that in the case of psychiatric PAD, an independent consultation from another expert is mandatory (5, 6).

Four years after Chabot's arrest, in 1998, the first guideline regarding PAD for psychiatric suffering was published by the Dutch Psychiatry Association. It contained detailed descriptions of many of the due diligence requirements that are still relevant today (see below). In 2001, the Dutch parliament voted in favor of the ‘Termination of Life on Request and Assisted Suicide Act’ that firmly established and clarified the due diligence procedures for PAD that had developed over the previous years. These legal requirements will be discussed in greater detail below. The Act became law in 2002 and at the time, the Dutch Minister of Health, Els Borst, emphasized that psychiatric suffering could also be ground for PAD. This historic legislative change was followed by a mostly quiet decade in the field of PAD for psychiatric suffering. The number of reported cases remained very low and the debate appeared dormant. The guideline underwent minor revisions by the Dutch Psychiatry Association in both 2004 and 2009, mainly regarding procedural demands.

**Figure 1:**
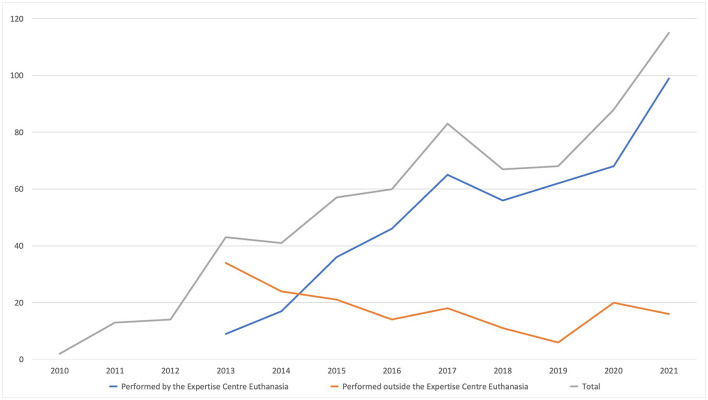
Number of physician-assisted deaths performed inside and outside of the Euthanasia Expertise-Center.

This relatively uneventful period ended when, in 2011, the number of reported cases started to rise (Figure 1). This rise coincided with the foundation of The End-of-Life Clinic; an organization specifically aimed at patients who wanted PAD but could not find support from their own physician, who could be opposed to PAD in general or in their specific case. The End-of-Life Clinic has quickly become the center of psychiatric PAD in the Netherlands. In the years following its establishment, an increasing portion of all reported assisted deaths for psychiatric suffering have been performed by physicians and psychiatrists working in The End-of-Life Clinic (Figure 1). The End-of-Life Clinic considered this problematic and, in 2019, renamed themselves as the *Expertise Center Euthanasia (ECE)* and started aiming more on supporting regular psychiatrists in performing PAD themselves. With the rising number of cases, societal attention and academic interest in the practice returned. This led to a major revision of the psychiatric PAD-guideline for in 2018, which we will further discuss below.

## Rules and Regulations

In the Netherlands, assisting in the death of another person is punishable by law. However, if physicians follow due care criteria as described in the ‘Termination of Life on Request and Assisted Suicide Review Act’ they are exempt from prosecution. From this point on we will refer to this act as the ‘Euthanasia Act.’ Both assisted suicide and euthanasia fall under the act, and a clinical guideline outlines medical procedures that should be followed and due care criteria that should be observed. In the case of assisted suicide, the patient ingests a fluid with a heavy sedative that will suppress the respiratory stimulus and stop the heart in a matter of min to h. In euthanasia, the physician intravenously administers a sedative that induces a coma, and consequently a muscle relaxant is given after which death follows almost immediately (7).

The six ‘due care’ criteria in the euthanasia act are the following. The physician must: (1) be satisfied that the patient's request is voluntary and well-considered; (2) be satisfied that the patient's suffering is unbearable and that there is no prospect of improvement; (3) inform the patient of his or her situation and further prognosis; (4) discuss the situation with the patient and come to the joint conclusion that there is no other reasonable solution; (5) consult at least one other physician with no connection to the case, who must then see the patient and state in writing that the attending physician has satisfied the due care criteria listed in the four points above; (6) exercise due medical care and attention in terminating the patient's life or assisting in his/her suicide.

When it concerns psychiatric suffering, an additional due care requirement applies. Based on jurisprudence and guidelines, a second opinion must be performed by an appropriate expert. This will usually be a psychiatrist working in an academic setting who specializes in the disorder the patient is suffering from (8).

To support patients and physicians throughout this challenging and long process, a guideline has been drawn up by the Dutch Psychiatric Association; the first version was published in 1998, the current version is from 2018 (9). The guideline distinguishes four phases: the request phase, the assessment phase, the consultation phase, and the implementation phase. The request phase begins when a patient expresses a wish for euthanasia. Important goals at that time are: to create an open and safe atmosphere in which to discuss the death wish, to carry out an assessment of possible acute suicidality, to check whether the relatives of the patient are aware of the request, and to provide information about the extensive euthanasia procedure that may follow. In the assessment phase, the physician assesses all due care criteria and requests the mandatory second opinion from an appropriate expert. In the consultation phase, a second physician is consulted. This is a physician, often a general practitioner, who normally has received specific training in assessing PAD-requests. These physicians are organized within a division of the Dutch Medical Association called ‘Support and Consultation for Euthanasia in the Netherlands’ and are therefore called SCEN-physicians. Finally, in the implementation phase, the assisted death takes place according to the proper procedure (7).

The Euthanasia Act also specifies how the PAD-procedure should be evaluated after the patient's death. Because it concerns an unnatural death, the body should be examined by a coroner directly. The physician who performed the PAD must report to a regional euthanasia oversight committee. In practice, this means that the physician fills out a standardized form describing how the due diligence demands were followed, accompanied by relevant medical correspondence, the reports of the independent physicians and the coroner's report. The regional euthanasia oversight committee exists of three members: a physician, an ethicist and a lawyer. If the oversight committee is satisfied that all due diligence demands were adequately followed, the physician is discharged from further prosecution. If there are doubts, the physician is sometimes asked to appear before the committee to provide additional information. If there are serious doubts about the legality of the PAD, the oversight committees can transfer the case to the public prosecutor for further investigation and possibly prosecution (8).

## Epidemiology

Although the past decade has seen a remarkable increase in PAD for psychiatric suffering, it remains relatively rare. In 2020, the total number of PADs was 6938, in 4480 cases the suffering was due to cancer (64.6%) and in 88 cases (1.3%) the suffering was due to a psychiatric disorder (2). However, the number of requests based on psychiatric suffering is much higher than the number that is actually performed. It is estimated that 56% of all Dutch psychiatrists have had a request for euthanasia during their career, and that about 95% of all requests are rejected (9, 10).

Detailed quantitative empirical research into PAD for psychiatric suffering, until recently, remained scarce. For many years, the only available source for research were the case summaries that were occasionally published on the website of the Dutch Regional Euthanasia Oversight Committees. Kim et al. studied 67 summaries of patients with a psychiatric disorder that received PAD between 2011 and 2014 (11). Van Veen et al. followed up on this research using the summaries that were published between 2015 and 2017 (12). Both studies found that most patients were diagnosed with multiple psychiatric disorders (71–79%). Common diagnoses were depression (46–74%), personality disorders and personality problems (52–54%), anxiety disorders (11–23%), and PTSD (20–23%). These studies were limited by the relatively low number of publicly available cases and the fact that they only concerned patients receiving PAD, not patients *requesting* PAD. This limitation does not apply to a report by the ECE that was presented at the beginning of 2020 (10). For this report, 1,308 files of patients who requested PAD for psychiatric suffering were analyzed. The report, that has not been peer reviewed or published in a scientific journal, shows that patients requesting PAD often have severe and long-standing psychiatric complaints, with 60% having a treatment history of more than 10 years. The mean age of applicants was 50. Sixty percentage of the applicants were women, 70% were single, 76% had a low or secondary education level and 88% were receiving benefits. 70% of the applicants had more than one psychiatric diagnosis. The most common main diagnosis was depression (35%). When comorbidity is taken into account, common diagnoses were depression (50%), cluster B personality disorder (22%) and trauma- and stressor-related disorders (20%). Almost 90% of the PAD requests due to psychiatric suffering did not end in PAD: 20% withdrew the request and 68% were rejected. Patients with a cluster B personality disorder as the main diagnosis were frequently rejected. The study also mentions eight patients who died by suicide after their request for PAD was rejected. Most patients whose PAD request was granted were between the ages of 50 and 60, 28% had a diagnosis of major depressive disorder and 13% a trauma- or stressor-related disorder.

## Current Challenges

### Irremediability

As mentioned above, Dutch law requires that there is no prospect of improvement and that there are no other solutions available before PAD is allowed. These legal demands align with the more universal view that irremediability of suffering is an important prerequisite for PAD (13). However, when we apply the concept of irremediability to psychiatric suffering, several challenges arise (14). First of all, most psychiatric disorders, with the possible exception of serious eating disorders, are not in themselves fatal. This means that patients with untreatable mental illnesses potentially have decades to live in which they can recover spontaneously or in which new treatment options can be developed that can still lead to recovery (15). Secondly, it is very difficult to give a reliable prognosis for psychiatric suffering, even for patients who have extensive treatment histories (16, 17). This has different reasons: little is known about the etiology of psychiatric suffering, there are hardly any reliable biomarkers, descriptive diagnostic reliability is relatively low and it is difficult to predict the effects of treatment (18). Thirdly, there is disagreement about whether patients who refuse certain treatments can be said to suffer irremediably. This is not a mere theoretical discussion: a casefile study shows that 56% of Dutch patients who received PAD due to psychiatric suffering did refuse some sort of treatment (11). Some authors argue that a patient must have tried all possible treatments before suffering can be seen as irremediable (19). This does however raise the question how effective imposed treatment can be in this specific context, especially when it concerns psychotherapy (14). Therefore, some authors state that there should be room for reasonable treatment refusal, or that only treatments patients consent to should be tried (20, 21). Whether the challenges concerning establishing irremediability are sufficient to prohibit PAD for psychiatric suffering is a matter of debate around the world (17, 22). A survey among 248 Dutch psychiatrists, showed that 56% thought it possible to establish irremediability. This implies that 44% doubted the possibility or thought establishing irremediability impossible, indicating dissensus among Dutch experts with real life experience in establishing irremediable psychiatric suffering (IPS).

Recent empirical studies provide directions for clinicians and policy makers on how to deal with these challenges. First, a qualitative interview study showed that Dutch psychiatrists with experience in establishing IPS in the context of PAD address these challenges by focusing on retrospective elements (18). Therefore, when establishing IPS, they do not necessarily assert that suffering will continue indefinitely, but more that the patient has done everything that reasonably can be asked to reduce suffering. Also, because treatment history plays an important role in this assessment, this means that it is not possible to establish IPS when a patient refuses substantial treatment. In another study, 53 experienced Dutch and Belgian psychiatrists were asked to reflect on potential criteria for IPS in the context of PAD (23). They agreed that a proper psychiatric diagnosis, that takes into account contextual factors, is necessary. All indicated biological and psychological treatments should have been tried, at least one recovery-oriented treatment should have been tried and if necessary substantial efforts should have been made to improve the patient's social situation. They also agreed that the complaints should be present for at least a few years, if only to give all these treatments a chance. Finally, this group agreed that there should also be limits to the number of treatments and diagnostic procedures a patient should undergo before IPS can be established.

### Decision-Making Capacity

Next to irremediability, questions about decision-making capacity play a central role in the debate about PAD for psychiatric suffering. Currently, all countries that allow a form of PAD require that a patient has the capacity to make an informed request (1). Decision-making capacity can be defined as the ability of an individual to make their own health care decisions (24). When assessing the capacity of a patient the physician must always relate it to a specific choice. In the context of PAD, the choice at hand is between life and death, which justifies a careful capacity review procedure. But even with a rigorous procedure in place, it remains challenging to assess decision-making capacity when a patient requests PAD for psychiatric suffering. First, research shows, that many patients requesting PAD suffer from long lasting and complex psychiatric disorders (10–12). This chronicity, may make it more challenging to assess if the death wish is unduly influenced by the psychiatric disorder (25). Second, authors worry about the influence of cognitive distortions, such as a ‘sense of burdensomeness’, on decision-making capacity in the context of a psychiatric PAD request (26).

The complexity of assessing capacity when patients request PAD due to psychiatric suffering is seen by different authors as a justified reason for precluding psychiatric suffering as a basis for PAD (17, 19). Other authors consider this blanket ban to be unjustified for two main reasons. First, it is argued that the group of patients with a mental disorder that actually have the capacity to make a PAD-request are 'sentenced' to further suffering by this exclusion (22). Second, others argue that cognitive distortions and irrational health beliefs should be assessed for every patient requesting PAD, as they are also present in patients suffering from somatic disorders (27). Empirical research on capacity in the context of psychiatric PAD is scarce. Studies of Dutch casefile summaries show that all patients who received PAD on the basis of psychiatric suffering were ultimately considered capable of making an informed decision (11, 12). One study showed that in 8% of the cases the doctors involved had differences of opinion about patient decision-making capacity, but in another study no cases of divergent views were found. However, some researchers are concerned about the rigor of the capacity assessments in the Netherlands, since many reports only contained a short statement about the patient possessing decision-making capacity, but lacked a description on how this was assessed (28). Questionnaire research among Dutch psychiatrists shows that 65% think they can determine whether a patient with a psychiatric disorder is capable of making a competent PAD-request, 12% think not, 23% have doubts (9). Empirical research into the decision-making capacity of patients requesting physician assisted death due to psychiatric suffering, for instance measured with the McArthur Competence Assesment Tool, could provide more clarity, but is not yet available.

## Conclusion

Physician assisted death for patients with a psychiatric disorder is a topic of increasing interest around the world. In the Netherlands, PAD has been an option for patients with a psychiatric disorder for decades and the practice is still evolving. The subject of PAD for patients with a psychiatric disorder is complex and controversial. Therefore, it is especially important that the debate about this topic is based on facts and is conducted with an openness for each other's viewpoints. The goal of this article is to contribute to an informed worldwide constructive dialogue on PAD for patients with a psychiatric disorder by making Dutch knowledge sources available to an international readership. We hope that this will help to further an informed and constructive debate on the topic.

## Data Availability Statement

The original contributions presented in the study are included in the article/supplementary material, further inquiries can be directed to the corresponding author/s.

## Author Contributions

SV performed the literature search. SV, GW, AB, and NE contributed to the writing of the article and approved the submitted version.

## Conflict of Interest

The authors declare that the research was conducted in the absence of any commercial or financial relationships that could be construed as a potential conflict of interest.

## Publisher's Note

All claims expressed in this article are solely those of the authors and do not necessarily represent those of their affiliated organizations, or those of the publisher, the editors and the reviewers. Any product that may be evaluated in this article, or claim that may be made by its manufacturer, is not guaranteed or endorsed by the publisher.

## Footnotes

^1^Parts of this article are also translated from the following Dutch publication: SMP? van Veen & GAM Widdershoven, Euthanasie in de psychiatrie. *Ned. Tijdschr. Geneeskd*. (2020).

## References

[1] Nicolini ME Kim SYH Churchill ME Gastmans C. Should euthanasia and assisted suicide for psychiatric disorders be permitted? A systematic review of reasons Psychol Med. (2020) 50:1241–56. 10.1017/S0033291720001543 32482180

[2] *Dutch Regional Euthanasia Oversight Committees - Annual Report 2021*. Available online at: https://www.euthanasiecommissie.nl/uitspraken/jaarverslagen/2021/maart/31/jaarverslag-2021

[3] van Ree F. Euthanasie en hulp bij zelfdoding in een psychiatrisch ziekenhuis. Med Contact. (1983) 6:749–53.

[4] van Pinxten P. Menswaardig sterven voor psychiatrische patiënten. (2012). Available online at: https://thesis.eur.nl/pub/11983/Pinxten,%20van%20P%20318351.pdf

[5] Schoevers RA Asmus FP Van Tilburg W. Physician-assisted suicide in psychiatry: Developments in the Netherlands. Psychiatr Serv. (1998) 49:1475–80. 9826251 10.1176/ps.49.11.1475

[6] Berghmans R. Commentary on “Suicide, euthanasia, and the psychiatrist”. Philos Psychiatry, Psychol. (1998) 5:131–5.

[7] *Guideline: Uitvoering euthanasie en hulp bij zelfdoding*. (2012). Available online at: https://www.knmp.nl/richtlijnen/uitvoering-euthanasie-en-hulp-bij-zelfdoding

[8] Regional Euthanasia Review Committess RTE: Code of Practice. The Netherlands (2015). 10.1017/CBO9781107415324.004

[9] Onwuteaka-Philipsen BD Legemaate J van der Heide A van Delden H Evenblij K El Hammoud I. Derde evaluatie Wet toetsing levensbeëindiging op verzoek en hulp bij zelfdoding. (2017). 16223080

[10] Kammeraat M Kölling P. Psychiatrische patiënten bij Expertisecentrum Euthanasie. (2020).

[11] Kim SYH de Vries R Peteet JR. Euthanasia and assisted suicide of patients with psychiatric disorders in the Netherlands 2011 to 2014. JAMA psychiatry. (2016) 73:362-8. 10.1001/jamapsychiatry.2015.2887 26864709 PMC5530592

[12] van Veen SMP Weerheim FW Mostert M van Delden JJM. Euthanasia of Dutch Patients with Psychiatric Disorders between 2015 and 2017. Tijdschr Psychiatr. (2018) 61:241–247. 31017282

[13] Widdershoven G Beekman A Evans N van Veen S. The Role of Suffering in the “Tired of Life” Debate. Am J Bioeth. (2022) 22:68–70. 10.1080/15265161.2021.2013981 35089841

[14] van Veen SMP Ruissen AM Widdershoven GAM. Irremediable psychiatric suffering in the context of physician-assisted death: a scoping review of arguments: la souffrance psychiatrique irrémédiable dans le contexte du suicide assisté: une revue étendue des arguments. Can J Psychiatry. (2020) 65:593–603. 10.1177/0706743720923072 32427501 PMC7457463

[15] Kirby J. Medical assistance in dying for suffering arising from mental health disorders: could augmented safeguards enhance its ethical acceptability? J Ethics and Mental Health. (2017) 10:1–18. Available online at: https://jemh.ca/issues/v9/documents/JEMH%20final%20Legislation-iv.pdf

[16] Fusar-Poli P Hijazi Z Stahl D Steyerberg EW. The science of prognosis in psychiatry: a review. JAMA Psychiatry. (2018) 75:1289–97. 10.1001/jamapsychiatry.2018.2530 30347013

[17] Blikshavn T Husum TL Magelssen M. Four reasons why assisted dying should not be offered for depression. J Bioeth Inq. (2017) 14:151–7. 10.1007/s11673-016-9759-4 27933459

[18] van Veen SMP Ruissen AM Beekman ATF Evans N Widdershoven GAM. Establishing irremediable psychiatric suffering in the context of medical assistance in dying in the Netherlands: A qualitative study. CMAJ. (2022) 194:E485–91. 10.1503/cmaj.210929 35273025 PMC8985907

[19] Appelbaum PS. Physician-assisted death in psychiatry. World Psychiatry. (2018) 17:145–6. 10.1002/wps.20548 29856553 PMC5980525

[20] Dembo J Schuklenk U Reggler J. “For Their Own Good”: a response to popular arguments against permitting Medical Assistance in Dying (MAID) where mental illness is the sole underlying condition. Can J Psychiatry. (2018) 63:451–6. 10.1177/0706743718766055 29635929 PMC6099778

[21] Berghmans R Widdershoven G Widdershoven-Heerding I. Physician-assisted suicide in psychiatry and loss of hope. Int J Law Psychiatry. (2013) 36:436–43. 10.1016/j.ijlp.2013.06.020 23830024

[22] Rooney W Schuklenk U van de Vathorst S. Are concerns about irremediableness, vulnerability, or competence sufficient to justify excluding all psychiatric patients from medical aid in dying? Heal Care Anal. (2017) 26:326–43. 10.1007/s10728-017-0344-8 28624976

[23] van Veen SMP Evans N Ruissen AM Vandenberghe J Beekman ATF Widdershoven GAM. Irremediable psychiatric suffering in the context of medical assistance in dying: A delphi-study. Canad J Psychiatry. (2022). 10.1177/07067437221087052. [Epub ahead of print]. 35311599 PMC9510999

[24] Charland LC. Decision-making capacity. (2008)

[25] Widdershoven GAM Ruissen A van Balkom AJLM Meynen G. Competence in chronic mental illness: the relevance of practical wisdom. J Med Ethics. (2017) 43:374–8. 10.1136/medethics-2014-102575 27165839

[26] Stoll J Ryan CJ Trachsel M. Perceived burdensomeness and the wish for hastened death in persons with severe and persistent mental illness. Front Psychiatry. (2021) 11:1482. Available online at: https://www.frontiersin.org/article/10.3389/fpsyt.2020.532817 33510652 10.3389/fpsyt.2020.532817 PMC7835407

[27] Dembo J van Veen S Widdershoven G. The influence of cognitive distortions on decision-making capacity for physician aid in dying. Int J Law Psychiatry. (2020) 72:101627. 10.1016/j.ijlp.2020.101627 32950802

[28] Doernberg SN Peteet JR Kim SYH. capacity evaluations of psychiatric patients requesting assisted death in the Netherlands. Psychosomatics. (2016) 57:556–65. 10.1016/j.psym.2016.06.005 27590345 PMC5097685

